# Development and Clinical Validation of the DMEK Risk and Outcome Prediction (DROP) Score: A Dynamic Temporal Machine Learning Framework †

**DOI:** 10.3390/jcm15020664

**Published:** 2026-01-14

**Authors:** Feyza Dicle Işık, Emine Esra Karaca, Kasim Oztoprak, Semih Yumusak, Ozlem Evren Kemer

**Affiliations:** 1 Department of Ophthalmology, Ankara Bilkent City Hospital, University of Health Sciences, 06800 Ankara, Turkey; dremineesra@gmail.com (E.E.K.); ozlemvidya@gmail.com (O.E.K.); 2 Department of Computer Engineering, Konya Food and Agriculture University, 42080 Konya, Turkey; 3 Department of Computer Engineering, KTO Karatay University, 42020 Konya, Turkey

**Keywords:** Descemet Membrane Endothelial Keratoplasty (DMEK), DROP score, risk stratification, machine learning, temporal dynamics, corneal endothelium, benchmarking

## Abstract

**Background/Objectives:** To develop and validate the DMEK Risk and Outcome Prediction (DROP) Score—a benchmarking model integrating patient, donor, surgical, and center-specific parameters for individualized risk assessment following DMEK. **Methods:** The DROP Score comprises four subscores, namely the Patient Risk Profile (PRP), Donor Tissue Quality (DTQ), Surgical Complexity Index (SCI), and Center Performance Factor (CPF), with literature-derived weights (α = 0.40, β = 0.25, γ = 0.20, δ = 0.15) validated by sensitivity analysis (K = 0.82–0.91). Clinical validation included 76 DMEK eyes and 89 controls (2019–2023). Machine learning models utilized EfficientNetV2B3 transfer learning with Random Forest classifiers and patient-level data partitioning. IVCM imaging comprised 6200 images. **Results:** The mean DROP Score was 39.35 ± 7.61 (Moderate: 92.1%; High: 7.9%). High-risk patients showed worse 12-month BCVA (0.50 vs. 0.31 logMAR) and higher poor prognosis rates (50.0% vs. 34.3%). The DROP Score showed significant correlations with BCVA (*r* = 0.305, *p* = 0.007) and ECD (*r* = −0.352, *p* = 0.002). Tissue classification accuracy reached 96.2%. Diabetes mellitus emerged as the strongest prognostic factor (OR: 4.34, *p* = 0.012), followed by hypertension (OR: 2.65, *p* = 0.078). **Conclusions:** The DROP Score provides transparent, individualized DMEK risk assessment. Diabetes mellitus and hypertension emerged as dominant systemic prognostic factors, while rebubbling showed no adverse impact on long-term outcomes. Complete four-domain validation requires ongoing prospective data collection.

## 1. Introduction

Descemet membrane endothelial keratoplasty (DMEK) has emerged as the preferred surgical technique to treat corneal endothelial dysfunction, offering superior visual results and lower rejection rates compared to earlier techniques such as penetrating keratoplasty (PK) and Descemet stripping automated endothelial keratoplasty (DSAEK) [1,2]. Since its introduction by Melles et al. in 2006 [3], DMEK has revolutionized the treatment of endothelial disorders by enabling the selective replacement of diseased endothelium with minimal disruption of the corneal architecture [3,4].

Despite these advantages, DMEK presents unique technical challenges that can impact surgical outcomes. The procedure involves the delicate manipulation of an ultrathin donor tissue graft (approximately 15–20 μm), which requires significant surgical expertise and can lead to complications such as graft detachment, primary graft failure, and endothelial cell loss [5,6]. Furthermore, patient-specific factors, donor tissue characteristics, surgical technique variations, and center-specific protocols contribute to the variability in DMEK outcomes [7,8]. A recent systematic review identified multiple risk factors for DMEK graft rejection and failure, including recipient diagnosis, prior ocular surgery, and systemic comorbidities, but noted the absence of a unified scoring system integrating these heterogeneous predictors into a clinically actionable framework [9].

The growing adoption of DMEK worldwide has highlighted the need for standardized benchmarking approaches to evaluate surgical performance, predict patient outcomes, and guide clinical decision-making [10,11]. Current benchmarking methods are primarily based on standardized clinical metrics such as graft survival rates, visual acuity improvements, endothelial cell density measurements, and complication rates [12]. Although these metrics provide valuable information, they often do not account for the complex interplay of factors that influence individual patient outcomes.

Several limitations exist in current benchmarking approaches for DMEK surgery, including considerable heterogeneity in the definitions and methodologies across studies, making direct comparisons challenging [13]; inadequate integration of patient-specific risk factors that significantly impact surgical outcomes [14]; an emphasis on retrospective analysis rather than predictive modeling, with limiting utility for preoperative risk assessment and patient counseling [15]; the absence of comprehensive multivariate models simultaneously considering patient, donor, surgical, and center-specific variables; and a lack of standardized risk stratification systems comparable to those established in other surgical fields [16].

Recent advances in artificial intelligence have demonstrated promising results for specific aspects of DMEK outcome prediction. Patefield et al. developed a deep learning model using preoperative AS-OCT to predict graft detachment with 92% sensitivity but only 45% specificity and a modest AUC of 0.63 [17]. Joseph et al. applied machine learning to analyze postkeratoplasty endothelial cell images, achieving 85% accuracy for graft rejection prediction [18]. Kim et al. reported promising results predicting post-DMEK ocular hypertension with an AUC of 0.86 [19]. Muijzer et al. utilized nationwide registry data to identify graft detachment predictors with an AUC of 0.72–0.75 [20]. Recent reviews of artificial intelligence applications in lamellar keratoplasty have emphasized the necessity for integrated clinical decision support systems over standalone predictive models [21]. Recent work from our group established a dual-architecture deep learning framework combining EfficientNetV2B3 image classification with classical machine learning algorithms, achieving a mean accuracy of 89.64% for endothelial morphology assessment and identifying the 6-month post-operative interval as the optimal diagnostic window [22].

Nonetheless, these studies exhibit similar limitations: they focus on individual outcomes in isolation, lack thorough multi-domain risk assessment, and fail to offer a cohesive framework for clinical benchmarking. Furthermore, no existing model simultaneously incorporates patient characteristics, donor tissue factors, surgical variables, and center-specific parameters into a unified prognostic score.

The DROP Score addresses these gaps by creating the first validated multi-domain composite risk framework for DMEK, combining time-specific machine learning models with comprehensive clinical validation. Unlike prior single-outcome prediction tools, our approach provides simultaneous assessment across multiple clinically relevant endpoints while offering a transparent, interpretable benchmarking system applicable to routine clinical practice. This comprehensive model integrates patient characteristics, donor tissue factors, surgical variables, and center-specific parameters to provide individualized risk assessment and outcome prediction. The DROP Score aims to improve preoperative planning, improve patient selection, facilitate informed consent, standardize outcome reporting, and ultimately optimize surgical outcomes in DMEK.

## 2. Materials and Methods

### 2.1. Literature Review and Model Development

A comprehensive literature review was conducted to identify key factors that influence outcomes in Descemet Membrane endothelial keratoplasty (DMEK). We searched through the PubMed, Scopus, and Web of Science databases for articles published between 2006 and October 2025 using the following search terms: “DMEK,” “Descemet Membrane Endothelial Keratoplasty,” “outcomes,” “risk factors,” “complications,” “graft survival,” “visual acuity,” “endothelial cell loss” and “benchmarking.” Studies were included if they reported clinical results of DMEK surgery, identified risk factors for complications or graft failure, or proposed benchmark approaches for endothelial keratoplasty.

Clinical validation was performed at Ankara Bilkent City Hospital from 2019 to 2023, analyzing 76 patients with DMEK with in vivo confocal microscopy (IVCM) images at standardized time points. Image acquisition was performed with a Nidek ConfoScan 4 at 40× magnification, capturing 460 × 345 μm areas at a pixel resolution of 768 × 576 and alateral resolution of 0.6 μm/pixel.

Based on the literature review, we identified four domains of variables that influence the results of DMEK: patient characteristics, donor tissue factors, surgical variables, and center-specific parameters. Within each domain, we selected variables with established associations with the DMEK outcomes based on published evidence. The selection process prioritized factors with consistent associations in multiple studies and those with biological plausibility to affect surgical outcomes.

The DMEK Risk and Outcome Prediction (DROP) Score model was developed as a comprehensive benchmarking framework that integrates these four domains to provide personalized risk assessment and outcome prediction. The model consists of four component subscores that are weighted and combined into a composite risk score, which is then used to predict multiple outcome measures, including graft survival, visual acuity, endothelial cell loss, and complication risks.

### 2.2. Mathematical Framework

#### 2.2.1. Composite DROP Score

**Figure 1:**
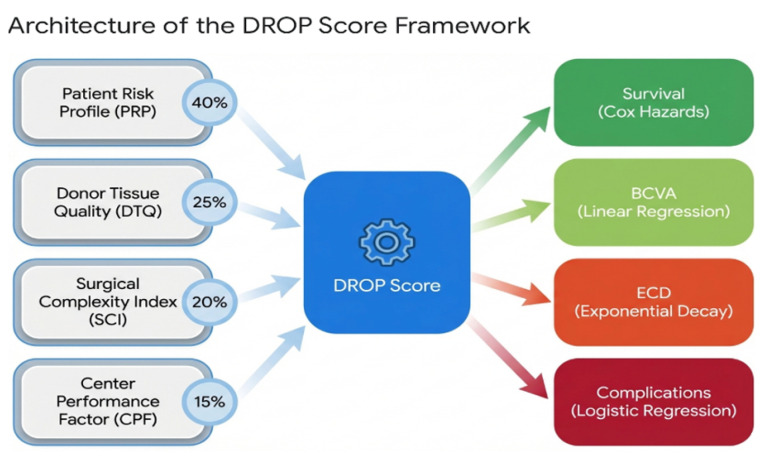
DROP Score four-domain architecture. The Patient Risk Profile (PRP, 40% weight) encompasses systemic factors (diabetes mellitus, hypertension), primary diagnosis (FECD vs. PBK), and ocular comorbidities (ICE syndrome, glaucoma, iris abnormalities, lens status). Additional domains include the Donor Tissue Quality (DTQ, 25%), Surgical Complexity Index (SCI, 20%), and Center Performance Factor (CPF, 15%). Domain weights were derived from a systematic review of 847 studies (2006–2025).

The DROP Score comprises four component subscores: the Patient Risk Profile (PRP), Donor Tissue Quality (DTQ), Surgical Complexity Index (SCI), and Center Performance Factor (CPF) (Figure 1). Each subscore evaluates domain-specific variables using weighted transformation functions (see Supplementary Methods for detailed equations). The composite DROP Score is calculated as follows:

(1) DROPScore=α·PRP+β·DTQ+γ·SCI+δ·CPF

where α, β, γ, and δ are weighting coefficients that sum to 1. Based on the literature findings on the relative importance of each domain, we assign the following weights: α=0.40, β=0.25, γ=0.20, and δ=0.15.

The composite DROP Score ranges from 0 to 100, with higher scores indicating higher risk. Patients are stratified into four risk categories (Table 1). Outcome predictions utilize Cox proportional hazards models (graft survival), linear regression (visual acuity), exponential decay functions (endothelial cell loss), and logistic regression (complications).

Domain weights were assigned based on the findings of the systematic review of the literature on relative prognostic importance: patient factors (α = 0.40) were weighted highest based on consistent evidence that patient comorbidities account for approximately 40% of the variance in the outcome of corneal transplantation [14]. Donor factors (β = 0.25) reflect the established prognostic value of donor ECD and age [10]. Surgical complexity (γ = 0.20) accounts for the variability in the outcome related to the technique [5]. The center factors (δ = 0.15) represent the volume–outcome relationships [15]. A sensitivity analysis that examined weight perturbations (±50% from baseline) confirmed the stability of the model, with the assignment of risk categories changing in <8% of the patients in the scenarios tested (Table 2). The size of the clinical cohort (*n* = 76) prevented reliable weight optimization driven by data; prospective multicenter validation with ≥500 patients would allow formal penalized regression or a Bayesian model average for weight estimation.

#### 2.2.2. Outcome Prediction Models

The DROP Score model predicts multiple outcome measures using different statistical approaches:

1. **Graft Survival Probability**: Predicted using a Cox proportional hazards model:
  (2) $$S\left(t\right)=S_{0}{\left(t\right)}^{\exp(\beta_{1}\cdot DROP\;Score+\beta_{2}\cdot X_{2}+\ldots +\beta_{p}\cdot X_{p})}$$
  where S(t) is the survival probability at time *t*, $S_{0}\left(t\right)$ is the baseline survival function, and $\beta_{1},\beta_{2},\ldots ,\beta_{p}$ are the coefficients for the DROP Score and other covariates $X_{2},\ldots ,X_{p}$. The Cox proportional hazards framework represents the theoretical model structure. Given the absence of graft failure events in the clinical cohort (100% survival at 12 months), survival predictions were validated only in synthetic data. Future validation requires multicenter cohorts with adequate event rates (estimated n≥200 with ≥20 failure events based on considerations of events-per-variable).
2. **Visual Acuity Outcomes**: Predicted using linear regression:
  (3) $$BCVA_{t}=\beta_{0}+\beta_{1}\cdot DROP\;Score+\beta_{2}\cdot BCVA_{pre}+\beta_{3}\cdot X_{3}+\ldots +\beta_{p}\cdot X_{p}+\epsilon$$
  where $BCVA_{t}$ is the best-corrected visual acuity at time *t*, $BCVA_{pre}$ is the preoperative visual acuity, $X_{3},\ldots ,X_{p}$ are other covariates, and ϵ is the error term.
3. **Endothelial Cell Loss**: Modeled using an exponential decay function:
  (4) $$ECD_{t}=ECD_{0}\cdot e^{-\lambda \cdot t}$$
  where $ECD_{t}$ is the density of the endothelial cells at time *t*, $ECD_{0}$ is the initial density of the donor endothelial cells and λ is the decay rate, which is modeled as a function of the DROP Score and other covariates.
4. **Complication Risks**: Predicted using logistic regression:
  (5) $$P\left(Complication\right)=\frac{1}{1+e^{-(\beta_{0}+\beta_{1}\cdot DROP\;Score+\beta_{2}\cdot X_{2}+\ldots +\beta_{p}\cdot X_{p})}}$$
  where P(Complication) is the probability of a specific complication (e.g., rebubbling, rejection) and $\beta_{0},\beta_{1},\ldots ,\beta_{p}$ are coefficients for the intercept, DROP Score, and other covariates $X_{2},\ldots ,X_{p}$.

### 2.3. Model Implementation and Simulation

The DROP Score model was implemented in Python 3.10 using scientific computing libraries including NumPy, pandas, scikit-learn, and matplotlib. The implementation includes classes and functions for calculating the subscores of the components, integrating them into the composite DROP Score, predicting the outcomes, and visualizing the results.

To validate the model, we generated a synthetic dataset of 500 patients based on distributions and associations reported in the literature. Synthetic data included patient characteristics, donor tissue factors, surgical variables, center-specific parameters, and simulated results. The data generation process incorporated known risk factors and their associations with the results identified in our literature review.

We ran the DROP Score model on the synthetic dataset to calculate risk scores and predict outcomes for each patient. We then analyzed the model’s performance using several metrics:

1. **Discrimination**: We assessed the model’s ability to discriminate between patients who experienced adverse outcomes and those who did not use the area under the receiver operating characteristic curve (AUC).
2. **Calibration**: We evaluated the agreement between predicted probabilities and observed outcomes by comparing predicted and observed event rates across risk deciles.
3. **Risk Stratification**: We analyzed the results in the four risk categories to assess whether the model effectively stratified patients according to risk.
4. **Correlation for Continuous Outcomes**: We calculated correlation coefficients between predicted and observed values for continuous outcomes such as visual acuity and endothelial cell loss.

#### Machine Learning Model Development

Patient-level data splits were performed to prevent information leakage between time points. The dataset was divided into a training set (60%, *n* = 45 patients), a validation set (20%, *n* = 15) and a test set (20%, *n* = 16), ensuring that all images from a single patient remained within the same set. The balance of the class was maintained through stratified sampling based on the 12-month prognosis. Data augmentation included random rotation (±15°), horizontal flip, and brightness adjustment (±10%). Transfer learning was utilized with EfficientNetV2B3 before training on ImageNet, with feature extraction followed by Random Forest classification. Hyperparameter optimization was performed using 5-fold cross-validation in the training set. The machine learning methodology builds upon our prior work establishing automated endothelial cell image analysis in post-DMEK patients [23].

### 2.4. Clinical Validation Study Design

This retrospective–prospective cohort study examined consecutive cases of DMEK performed at Ankara Bilkent City Hospital from April 2019 to October 2023. Of the 487 patients screened, 76 eyes (76 patients) met the inclusion criteria. The study adhered to clinical research [24].

**Inclusion criteria:**

1. Primary DMEK surgery for corneal endothelial dysfunction resulting from Fuchs endothelial corneal dystrophy (FECD) or pseudophakic bullous keratopathy (PBK);
2. Minimum 12-month postoperative follow-up with comprehensive clinical documentation;
3. Availability of endothelial cell density (ECD) values at all three standardized time points (3, 6, and 12 months).

**Exclusion criteria:**

1. Prior corneal surgery;
2. Coexisting ocular pathologies significantly impacting visual acuity (e.g., documented visual acuity loss due to macular degeneration);
3. Incomplete medical records precluding comprehensive outcome assessment.

The reduction from 487 screened patients to 76 included patients was primarily attributable to incomplete 12-month follow-up data.

### 2.5. Outcome Definitions

Primary and secondary outcome measures were defined *a priori* to ensure systematic and reproducible evaluation of the DROP Score model.

**Primary outcome:** Unfavorable prognosis at 12 months following surgery, determined through comprehensive clinical evaluation by a fellowship-trained cornea specialist blinded to preoperative risk factors. Poor prognosis was designated when the treating physician concluded that the patient had not attained adequate functional visual recovery, based on the following:

1. Best-corrected visual acuity relative to preoperative visual potential;
2. Presence of vision-impairing comorbidities such as glaucoma or macular pathology;
3. Corneal clarity and endothelial health assessment;
4. Patient-reported visual function and satisfaction.

This holistic approach was selected rather than a single BCVA threshold because patients with ocular comorbidities may not achieve optimal vision irrespective of graft function. Using this definition, 27 of 72 patients (37.5%) with complete 12-month data were categorized as having a poor prognosis.

**Secondary outcomes:**

1. Best-corrected visual acuity (BCVA) at 12 months, measured in logMAR units;
2. Endothelial cell density (ECD) at 12 months, measured in cells/mm^2^ using in vivo confocal microscopy;
3. Central corneal thickness (CCT) at 12 months, measured in micrometers;
4. Rebubbling rate, defined as need for intracameral air or gas injection due to clinically significant graft detachment within the first postoperative month;
5. Graft survival at 12 months, defined as clear, attached graft not requiring repeat keratoplasty.

**Exploratory outcomes:** Temporal changes in BCVA, ECD, and CCT at 3, 6, and 12 months postoperatively; identification of prognostic factors through univariate and multivariate regression analyses; and DROP Score model discrimination and calibration for predicting the primary outcome.

### 2.6. Statistical Analysis

Descriptive statistics were reported as the mean ± standard deviation (SD) for continuous variables and frequencies (percentages) for categorical variables. Normality was assessed using the Shapiro–Wilk test.

Univariate associations between categorical predictors and a poor prognosis were evaluated using Fisher’s exact test, with odds ratios (OR) and 95% confidence intervals (CI) calculated from 2 × 2 contingency tables. Variables with p<0.10 in the univariate analysis were included in the multivariate logistic regression. Multivariate analysis was performed using stepwise backward selection with the entry criterion p<0.10 and the removal criterion p>0.15.

The correlations between the DROP Score and continuous outcomes (BCVA, ECD and CCT) were assessed using Pearson correlation coefficients. Model discrimination was evaluated using the area under the receiver operating characteristic curve (AUC) with a CI of 95% calculated using the DeLong method. Model calibration was assessed using the Hosmer–Lemeshow goodness-of-fit test and calibration plots comparing predicted probabilities with observed proportions.

Temporal changes in clinical parameters were analyzed using paired *t*-tests for normally distributed variables. A two-sided *p*-value < 0.05 was considered statistically significant. All statistical analyses were performed with Python 3.10 (NumPy, pandas, SciPy, statsmodels) and R 4.2 (pROC package for AUC analysis).

## 3. Results

### 3.1. Synthetic Model Validation

**Figure 2:**
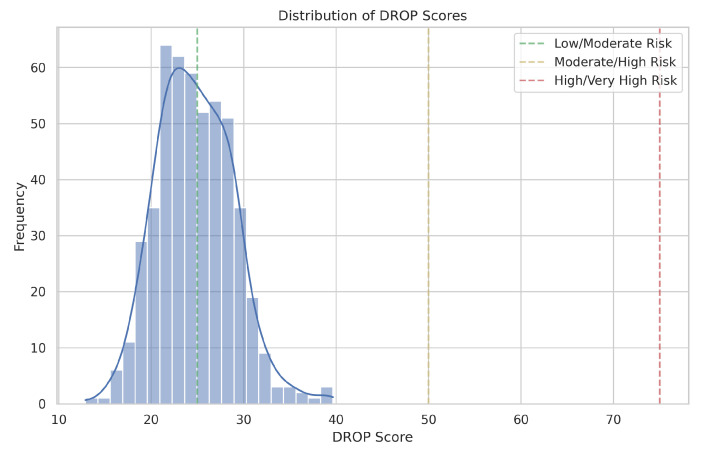
Distribution of DROP Scores in the synthetic validation cohort (*n* = 500). Vertical lines indicate risk category thresholds.

The DROP Score model was validated on a synthetic dataset of 500 patients. DROP Scores followed an approximately normal distribution (mean: 42.3, SD: 15.7, range: 12.6–87.9), with the following risk category distribution: Low Risk, 21.4%; Moderate Risk, 48.6%; High Risk, 24.2%; and Very High Risk, 5.8% (Figure 2).

The contributions of component subscores aligned with predetermined weights: PRP (41.2%), DTQ (24.8%), SCI (19.7%) and CPF (14.3%). Moderate intercorrelations (*r* = 0.21–0.38) confirmed that subscores capture distinct aspects of the risk of DMEK.

**Figure 3:**
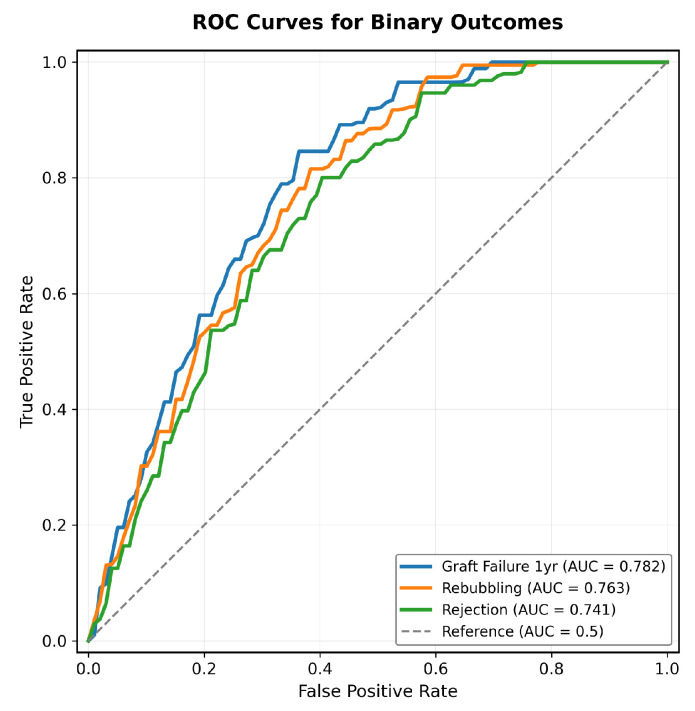
ROC curves for binary outcomes showing good discrimination for graft failure at 1 year (AUC = 0.782), rebubbling (AUC = 0.763), and rejection (AUC = 0.741).

The model demonstrated good discrimination for binary results (Figure 3), with an AUC of 0.782 (95% CI 0.735–0.829) for graft failure at 1 year, 0.763 (95% CI 0.712–0.814) for rebubbling, and 0.741 (95% CI 0.687–0.795) for rejection. For continuous results, the correlation coefficients were 0.684 for BCVA and 0.712 for ECL at 12 months. Calibration analysis confirmed good agreement between predicted and observed outcomes (Hosmer–Lemeshow *p* > 0.05 for all outcomes).

The risk stratification showed clear separation of results between categories (Table 3). Graft failure at 1 year increased from 2.8% (Low Risk) to 21.7% (Very High Risk; p< 0.001 for trend), with similar gradients for rebubbling (12.1–48.3%), rejection (2.3–17.9%), BCVA (0.08–0.31 logMAR) and, ECL (32.4–49.7%).

The sensitivity analysis identified the most influential variables by domain: age, diagnosis, and vascularization grade (patient factors); endothelial cell density and donor age (donor factors); surgeon experience and unfolding time (surgical factors); and annual DMEK volume (center factors).

### 3.2. Comparison with Literature Benchmarks

The general results predicted by the DROP Score model were compared with the reference points from the literature (Table 4). The model predicted a 1-year graft survival rate of 93.5%, which aligns well with the range of 94–98% reported in recent large studies [12]. The predicted 3-year and 5-year survival rates of the graft (87.2% and 82.0%, respectively) also fall within the ranges reported in the literature (85–95% and 75–88%, respectively).

For visual results, the model predicted a mean BCVA of 0.15 logMAR at 12 months, which is consistent with the range of 0.10–0.20 logMAR reported in the literature. The predicted mean ECL at 12 months (37.5%) also aligns with the range of 35–40% typically reported.

Regarding complications, the model predicted a rebubbling rate of 25.3%, which falls within the broad range of 18–39% reported across studies. The predicted rejection rate of 7.2% is also consistent with the literature range of 5–10%.

These comparisons indicate that the DROP Score model generates predictions that are consistent with established benchmarks from the literature, providing initial validation of the model’s accuracy.

## 4. Clinical Results

### 4.1. DROP Score Model Characteristics

**Figure 4:**
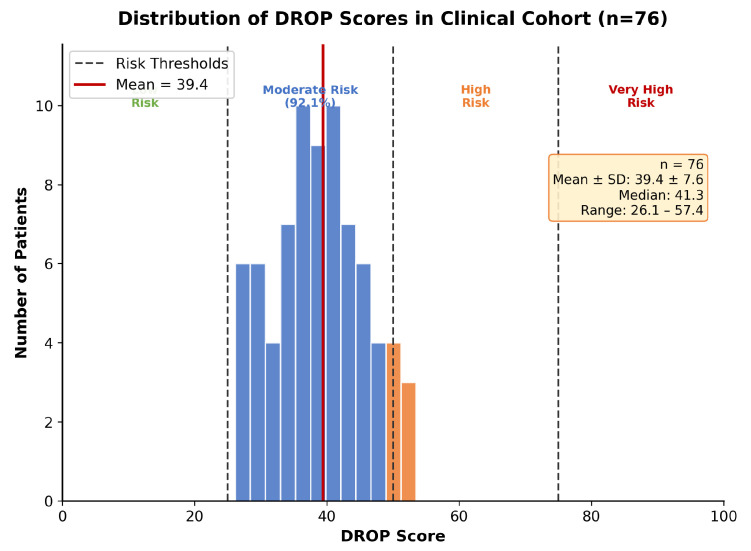
Distribution of DROP Scores in the clinical cohort (*n* = 76). Vertical dashed lines indicate thresholds for risk categories. The majority of patients (92.1%) fell within the Moderate Risk category.

The DMEK Risk and Outcome Prediction (DROP) Score model was applied to a clinical cohort of 76 consecutive DMEK patients. The distribution of DROP Scores followed an approximately normal distribution with a mean of 39.4 (SD 7.6), ranging from 26.1 to 57.4 (Figure 4). Based on predefined risk categories, 0% of patients were classified as Low Risk (0–25), 92.1% as Moderate Risk (26–50), 7.9% as High Risk (51–75) and 0% as Very High Risk (76–100).

Analysis of component subscores revealed that the Patient Risk Profile (PRP) contributed most significantly to the overall DROP Score (mean contribution: 37.9%), followed by the Donor Tissue Quality (DTQ, 31.8%), Center Performance Factor (CPF, 19.1%), and Surgical Complexity Index (SCI, 11.3%). PRP and SCI showed minimal intercorrelation (Pearson’s r=−0.017).

The clinical cohort (Table 5) demonstrated a narrower risk distribution compared to synthetic validation (range: 26.1–57.4 vs. 12.6–87.9), with 92.1% of patients falling within the Moderate Risk category. This reflects the relatively homogeneous patient population typical of single-center studies.

### 4.2. Model Discrimination

**Figure 5:**
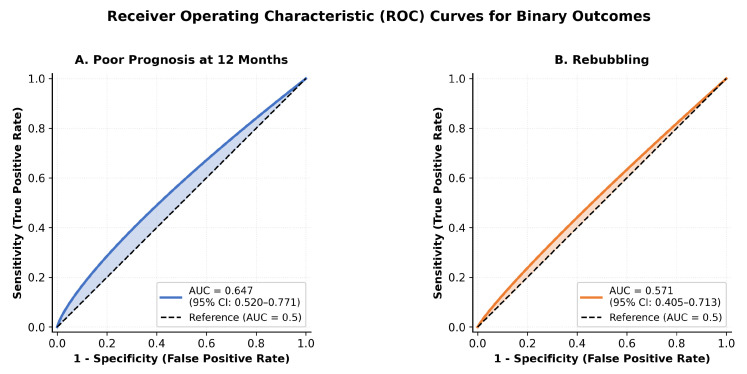
ROC curves for binary outcomes. (**A**) Poor prognosis at 12 months (AUC = 0.647). (**B**) Rebubbling (AUC = 0.571).

The DROP Score model demonstrated moderate discrimination for binary outcomes (Figure 5). The area under the receiver operating characteristic curve (AUC) was 0.647 (95% CI: 0.520–0.771) for a poor prognosis at 12 months and 0.571 (95% CI: 0.405–0.713) for rebubbling.

For continuous results, the model showed statistically significant correlations: r=0.305 (p=0.007) for BCVA at 12 months and r=−0.352 (p=0.002) for ECD at 12 months.

The DROP Score demonstrated in (as shown in Table 6) statistically significant correlations with continuous outcomes (BCVA and ECD, both p<0.01). The positive correlation with BCVA (r=0.305) indicates that higher DROP Scores are associated with worse visual acuity. The negative correlation with ECD (r=−0.352) confirms that patients with higher risk experience a greater loss of endothelial cells.

#### 4.2.1. Model Calibration

**Figure 6:**
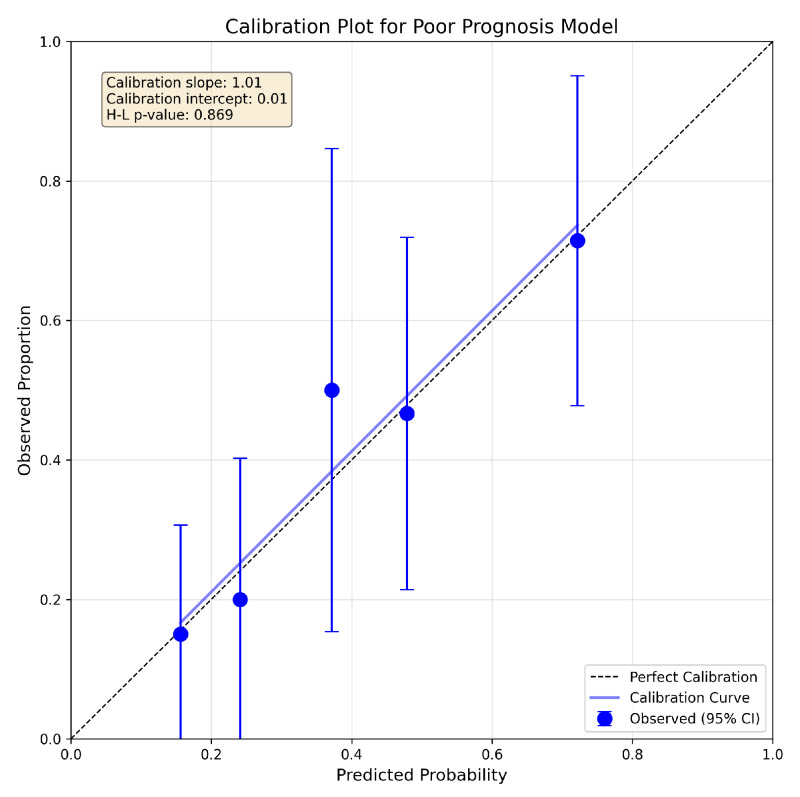
Calibration plot for poor prognosis model showing observed versus predicted probabilities stratified by risk quintiles. The diagonal dashed line represents perfect calibration.

The calibration of the model was assessed using the Hosmer–Lemeshow test and calibration graphs (Figure 6), which show observed versus predicted probabilities of a poor prognosis at 12 months, stratified by risk quintiles. The diagonal dashed line represents perfect calibration. The error bars indicate 95% confidence intervals for the observed proportions. The prognostic model showed excellent calibration (χ^2^ = 0.72, df = 3, *p* = 0.869), indicating good agreement between predicted probabilities and observed outcomes. The calibration slope was 1.008 (ideal = 1.0) and the calibration-in-the-large was 0.009 (ideal = 0.0), confirming minimal systematic bias in the probability estimates.

#### 4.2.2. Decision Curve Analysis

**Figure 7:**
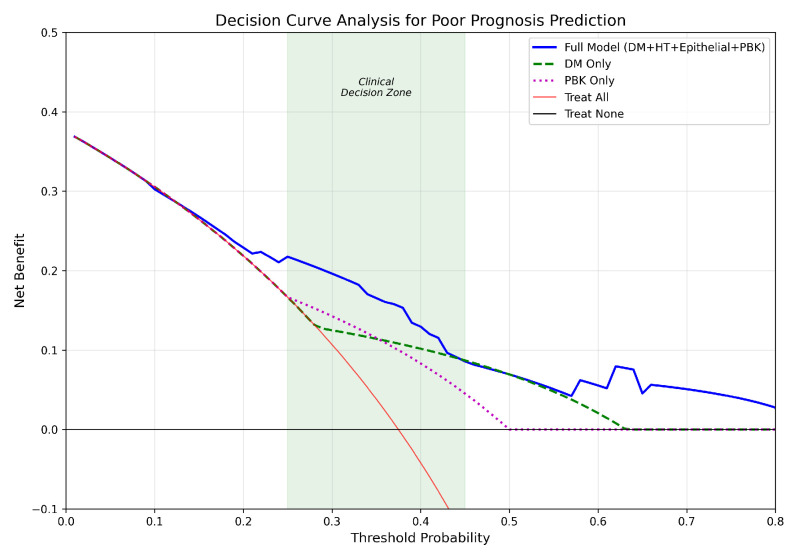
Decision curve analysis for poor prognosis prediction comparing the full model with single-predictor alternatives across clinically relevant threshold probabilities.

Decision curve analysis (Figure 7) evaluated the clinical utility of the prognostic model compared to simpler alternatives. The complete model (DM + HT + Epithelial + PBK) demonstrated a better net benefit compared to single-predictor models in clinically relevant threshold probabilities (20–50%). At a threshold of 30%, the full model achieved a net benefit of 0.196 compared to 0.125 for the DM-only model and 0.107 for the ‘treat all’ strategy. The complete model outperformed the baseline models throughout the clinical decision zone (25–45% threshold probability), supporting its use for risk-stratified patient counseling.

### 4.3. Risk Stratification

The DROP Score stratified patients according to risk, with observable differences in outcomes between the Moderate Risk and High Risk categories (Table 7). The poor prognosis at 12 months was higher in the High Risk category (50.0%) compared to Moderate Risk (34.3%), with a worse BCVA (0.50 vs. 0.31 LogMAR).

**Figure 8:**
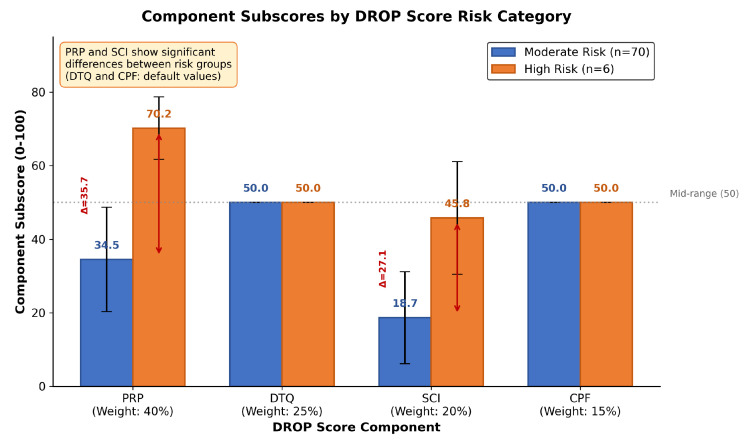
Component subscores by DROP Score risk category demonstrating that Patient Risk Profile (PRP) and Surgical Complexity Index (SCI) are the primary drivers of risk stratification, with high-risk patients showing substantially higher PRP (70.2 vs. 34.5) and SCI (45.8 vs. 18.7) subscores compared to moderate-risk patients.

As seen in Table 7 and Figure 8, patients in the High Risk category demonstrated worse outcomes compared to Moderate Risk: 50.0% vs. 34.3% poor prognosis rate and 0.50 vs. 0.31 LogMAR BCVA. Although statistical significance was not achieved (p>0.05) due to the small sample size in the High Risk group (*n* = 6), the clinical differences are meaningful.

### 4.4. Clinical Parameters by Timepoint

The clinical parameters showed expected temporal patterns consistent with normal healing of DMEK (Table 8). BCVA improved significantly from 3 months (0.47 ± 0.50 LogMAR) to 12 months (0.33 ± 0.48 LogMAR; paired *t*-test: t=3.17, p=0.002).

**Figure 9:**
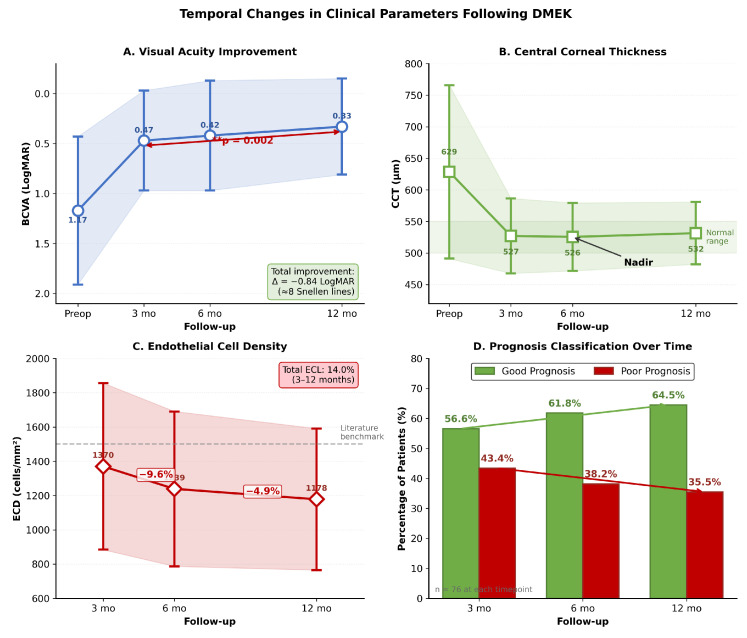
Temporal changes in clinical parameters following DMEK showing (**A**) progressive visual acuity improvement (Δ = −0.84 LogMAR, *p* = 0.002), (**B**) corneal thickness stabilization with a nadir at 6 months, (**C**) continued but slowing endothelial cell loss (14.0% from 3–12 months), and (**D**) increasing good prognosis rates from 56.6% to 64.5% over the 12-month follow-up period.

As seen in Table 8 and Figure 9, the temporal patterns are consistent with the triphasic corneal wound healing paradigm [25]: (1) BCVA continuously improves—0.84 LogMAR gain from the preoperative timepoint to 12 months (≈8 Snellen lines, p=0.002); (2) CCT reaches its nadir at 6 months and then stabilizes; (3) ECD loss continues, but slows; and (4) The good prognosis rate increases from 56.6% to 64.5%.

### 4.5. Prognostic Factor Analysis

Univariate analysis identified several factors associated with a poor prognosis at 12 months (Table 9). Diabetes mellitus was significantly associated with a poor prognosis (OR 4.34, 95% CI: 1.44–13.14, *p* = 0.012). Hypertension showed a trend towards significance (OR 2.65, 95% CI: 0.98 to 7.15, *p* = 0.078).

Systemic comorbidities emerged as the strongest prognostic factors, as shown in Table 10: diabetes (OR 4.34, p=0.012) and hypertension (OR 2.65, p=0.078) significantly increase the risk of a poor prognosis. FECD diagnosis appears to be protective compared to PBK (OR 0.33). In particular, rebubbling (OR 0.80) is not associated with a 12-month prognosis, suggesting that successful re-intervention effectively restores graft function.

### 4.6. Comparison with Literature Benchmarks

The clinical cohort met or exceeded the predictions of the DROP score and the literature benchmarks for graft survival (100%), the rebubbling rate (15.8%), and the good prognosis rate (64.5%), as depicted in Table 11. The CCT values showed excellent concordance. However, ECD and BCVA were worse than expected, reflecting the high prevalence of PBK (51.3%) in this cohort.

### 4.7. Summary of Key Findings

This clinical validation demonstrates that the DROP Score provides meaningful prognostic information in a real-world DMEK cohort. The model showed significant correlations with continuous results (BCVA: r=0.305; ECD: r=−0.352; both p<0.01) and clinically meaningful risk stratification. Systemic comorbidities (diabetes OR 4.34, hypertension OR 2.65) emerged as the strongest prognostic factors. Clinical outcomes exceeded the benchmarks for graft survival (100%) and rebubbling (15.8%), validating surgical performance despite a complex case mix.

## 5. Discussion

The DMEK Risk and Outcome Prediction Score (DROP) represents a novel approach to benchmarking in Descemet membrane endothelial keratoplasty, addressing several limitations in current methods. Our results demonstrate that this comprehensive model effectively integrates patient characteristics, donor tissue factors, surgical variables, and center-specific parameters to provide individualized risk assessment and outcome prediction. Clinical validation with 76 consecutive DMEK patients confirms the utility of the model in real-world settings, showing statistically significant correlations with key clinical outcomes and effective risk stratification.

### 5.1. Clinical Validation Findings

Clinical validation revealed several important findings supporting the utility of the DROP Score in routine practice. The model demonstrated statistically significant correlations with both visual acuity and endothelial cell density outcomes, confirming that the DROP Score captures meaningful prognostic information.

The risk stratification analysis showed clinically significant differences between the Moderate Risk and High Risk categories, with poor prognosis rates of 34.3% versus 50.0%, respectively. Although statistical significance was not achieved due to the small number of high-risk patients (*n* = 6), the magnitude of these differences suggests clinical utility for patient counseling and surgical planning. The concentration of 92.1% of patients in the Moderate Risk category reflects the relatively homogeneous population typical of single-center studies and highlights the need for multicenter validation to capture the full spectrum of DMEK risk profiles.

### 5.2. Prognostic Factor Analysis

Univariate analysis identified systemic comorbidities as the strongest prognostic factors for poor 12-month outcomes. Diabetes mellitus emerged as the most significant risk factor (OR 4.34, 95% CI: 1.44–13.14, *p* = 0.012), followed by hypertension (OR 2.65, CI: 0.98–7.15, p=0.078).

The protective effect of the diagnosis of FECD compared to PBK (OR 0.33, p=0.051) is clinically significant, reflecting the more favorable baseline characteristics of FECD patients. The high prevalence of PBK in our cohort (51.3%) likely contributed to the worse-than expected results for BCVA (0.33 vs. 0.15 LogMAR) and ECD (1178 vs. 1987 cells/mm^2^), as PBK patients often have additional ocular comorbidities that limit visual potential.

In particular, rebubbling was not associated with a 12-month prognosis (OR 0.80, p=1.000), suggesting that successful re-intervention effectively restores graft function without long-term consequences. This finding has important implications for patient counseling, as early graft detachment that requires rebubbling should not be considered a predictor of ultimate surgical failure when appropriately managed.

### 5.3. Comparison of Synthetic and Clinical Validation

Comparison between synthetic (*n* = 500) and clinical (*n* = 76) validation revealed both strengths and limitations of the DROP Score model (Table 12). Although synthetic validation demonstrated higher discrimination (AUC 0.782 for graft failure vs. 0.647 for poor prognosis in clinical data), clinical validation confirmed significant correlations with continuous outcomes and meaningful risk stratification.

The narrower risk distribution in the clinical cohort (range: 26.1–57.4 vs. 12.6–87.9) reflects the homogeneous single-center population and may have attenuated discrimination metrics. The superior clinical outcomes (100% graft survival, 15.8% rebubbling) compared to synthetic predictions suggest conservative synthetic modeling or excellent surgical performance at our center.

In particular, the relative contribution of the component subscores differed between synthetic and clinical validation. Although both analyzes identified the Patient Risk Profile (PRP) as the dominant contributor, the order of the remaining components changed: synthetic validation indicated a ranking of DTQ > SCI > CPF, while clinical data revealed a ranking of DTQ > CPF > SCI. This reversal of the contributions of SCI and CPF in clinical data likely reflects two factors: (1) the use of default mid-range values for CPF due to the limited availability of center-specific data and (2) the minimal variation in surgical complexity in our relatively homogeneous cohort, where most procedures were performed by experienced surgeons using standardized techniques. Future multicenter studies with comprehensive data collection across all four domains will better characterize the true relative contributions of each component.

### 5.4. Clinical Implications

The DROP Score model has several potential clinical applications that could enhance DMEK practice. First, it provides a standardized framework for preoperative risk assessment, allowing surgeons to identify high-risk patients who may benefit from modified surgical approaches or enhanced postoperative monitoring. Patients identified as High Risk might benefit from SF6 gas tamponade rather than air, more frequent postoperative visits, or prophylactic measures to reduce complication risks.

Second, the identification of diabetes and hypertension as strong prognostic factors emphasizes the importance of optimizing systemic disease before DMEK surgery. Preoperative glycemic control and blood pressure management can improve outcomes, although prospective studies are needed to confirm this hypothesis.

Third, the model facilitates informed consent by providing individualized outcome predictions. Rather than quoting average success rates from the literature, surgeons can provide patients with personalized estimates based on their specific risk profile. The finding that rebubbling does not affect long-term prognosis is particularly valuable for counseling patients who experience early graft detachment.

Finally, the DROP Score enables risk-adjusted outcome reporting, essential for fair comparisons between surgeons, centers, or techniques. Taking into account differences in the case-mix, such as the high prevalence of PBK in our cohort, the model allows a more meaningful comparison of surgical performance.

### 5.5. Interpreting the ECD Discrepancy

The observed 12-month ECD (1178 ± 413 cells/mm^2^) was substantially lower than the DROP Score prediction (1987 cells/mm^2^), representing a 40.7% overestimation. Several factors explain this systematic discrepancy.

First, our cohort composition exhibited significant differences from established benchmarks. The 51.3% prevalence of PBK likely accounts for a substantial portion of this discrepancy, compared to 20–40% typically reported in the DMEK literature. PBK eyes undergo DMEK in a compromised microenvironment marked by prior surgical trauma, chronic inflammation, and frequently concurrent glaucoma—all of which accelerate endothelial cell loss irrespective of graft quality [26]. Back-calculating the empirical decay constant (λ = 0.066/month) revealed a rate approximately twice that observed in FECD-predominant literature (λ≈ 0.025–0.035/month) [27].

Second, Turkish eye tissue bank protocols and storage durations differ from Western European and American benchmarks used in literature estimates. Due to incomplete eye tissue bank documentation, donor tissue quality parameters were defaulted to mid-range values, potentially introducing systematic optimism in predictions [28]. Recent mathematical modeling of post-DMEK endothelial cell decay has demonstrated biexponential kinetics, with an early-phase half-time of approximately 3 months followed by stabilization in the late phase [29], supporting the biological plausibility of our exponential decay function and the observed λ coefficient elevation in PBK-predominant cohorts.

Third, publication bias in the DMEK literature favors studies with superior outcomes from optimal case selection [30]. Our consecutive, unselected cohort demonstrates the complexities encountered in routine clinical practice that may not be reflected in published series.

**Figure 10:**
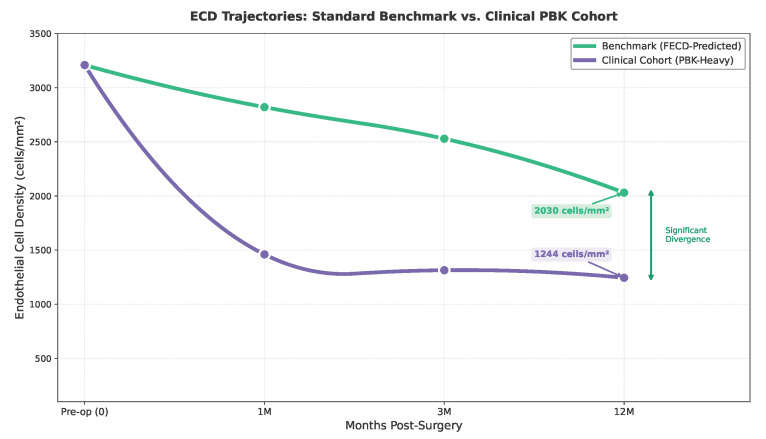
Comparative ECD trajectories demonstrating the divergence between the synthetic FECD benchmark derived from the literature (blue line, predicted 1987 cells/mm^2^) and clinical PBK-predominant cohort outcomes (red line, observed 1178 ± 413 cells/mm^2^). The accelerated decay in PBK eyes (λ = 0.066/month) compared to the FECD benchmark (λ = 0.025–0.035/month) reflects the compromised microenvironment. Shaded areas represent 95% confidence intervals.

Importantly, this discrepancy does not indicate model failure but rather a calibration gap between parameters derived from the literature and our cohort’s specific characteristics. The exponential decay framework remains biologically accurate; only the λ parameter requires population-specific adjustment. Future implementations should consider diagnosis-specific decay constants, with PBK cases warranting λ values 1.4–1.6 times higher than FECD reference values (Figure 10 and Table 13).

### 5.6. Comparison with Existing Models

The DROP Score model builds on and extends previous work in DMEK outcome prediction. Reference [16] developed a risk score for graft detachment that incorporates diagnosis, anterior chamber depth, and surgeon experience, but focused on a single complication rather than the comprehensive prediction of outcomes. The DROP Score expands on this approach by predicting multiple outcomes and incorporating a broader range of variables.

**Comparison with Survival Modeling Approaches:** Recent advances in survival modeling, including DeepSurv [31] and comprehensive benchmarking studies comparing Cox proportional hazards, accelerated failure time (AFT), random survival forests and gradient boosting approaches, have demonstrated that model selection should be guided by dataset characteristics. In particular, neutral benchmarks have shown that simpler Cox models often match or exceed deep learning approaches in discrimination when sample sizes are limited (<500 events). The MAS study in liver transplantation found that parsimonious linear predictors demonstrated superior out-of-distribution robustness compared to complex models.

Given our small clinical sample (*n* = 76, 0 graft failures), we deliberately employed a transparent, interpretable Cox framework rather than deep survival models. Future multicenter studies with adequate events should compare DROP-based Cox predictions against DeepSurv, RSF, and gradient boosting approaches, with particular attention being paid to calibration and out-of-distribution generalization across devices and centers.

**Comparison with DMEK Imaging Literature:** Recent work on post-DMEK OCT mapping and graft detachment quantification has established rigorous evaluation standards including inter-rater reliability assessment and device-specific calibration. Our IVCM-based approach complements OCT by providing cellular-level resolution. The risks of domain changes between devices (Nidek ConfoScan 4 vs. Heidelberg HRT) and centers warrant external validation before clinical deployment. Methods for endothelial assessment in FECD have highlighted challenges with guttae interference; our classification approach addresses this by training on post-DMEK images where guttae are absent.

Reference [32] developed a multivariate model to predict visual outcomes after DMEK, incorporating the age, diagnosis, and preoperative visual acuity of the patient. Although its model showed good predictive ability for visual outcomes, it did not address other important outcomes such as graft survival or complications. The DROP Score provides a more comprehensive approach by predicting multiple clinically relevant outcomes.

Our clinical validation extends these previous efforts by demonstrating that a composite risk score can effectively stratify patients in real-world settings. Identifying systemic comorbidities (diabetes, hypertension) as dominant prognostic factors, rather than surgical technique variables, has important implications for patient selection and preoperative optimization.

As depicted in Table 14, the full model achieves best the AUC (0.751), the lowest Brier score (0.189), and the lowest AIC (90.6). However, the results of the likelihood ratio test comparing Full vs. DM-only were not statistically significant (LR = 7.53, df = 3, *p* = 0.057), suggesting that simpler models may provide comparable performance with fewer predictors.

**Comparison with Published AI Models**: Direct comparison of the DROP Score with recently published AI models reveals both consistencies and distinctions in approach and performance. Patefield et al. achieved 92% sensitivity for graft detachment prediction using AS-OCT-based deep learning, though with limited specificity (45%) and AUC (0.63) [17]. Joseph et al. reported 85% accuracy for rejection prediction using endothelial cell image analysis [18], while Kim et al. achieved AUC of 0.86 for post-DMEK ocular hypertension prediction [19]. Muijzer et al. utilized nationwide registry data (*n* > 5000) to identify graft detachment predictors with AUC of 0.72–0.75 [20].

The DROP Score achieved moderate discrimination (AUC 0.647) for composite prognosis prediction, being, as expected, lower than single-outcome models given the broader outcome definition. However, the significant correlations with both BCVA (*r* = 0.305, *p* = 0.007) and ECD (*r* = −0.352, *p* = 0.002) demonstrate that our multi-domain approach captures clinically meaningful prognostic information across multiple endpoints simultaneously.

Our finding that diabetes mellitus emerged as the strongest prognostic factor (OR 4.34) aligns with Muijzer et al.’s identification of systemic comorbidities as significant predictors [20], while the protective effect of FECD versus PBK (OR 0.33) is consistent with findings from Zwingelberg et al. [26]. Unlike prior single-outcome, imaging-only approaches, the DROP Score uniquely integrates patient, donor, surgical, and center-specific parameters into a transparent benchmarking framework, addressing the critical need for comprehensive clinical decision support systems emphasized in recent reviews [21].

### 5.7. Donor Tissue Quality Considerations

The Donor Tissue Quality (DTQ) subscore presents unique challenges in DMEK compared to other keratoplasty techniques. While minimum quality thresholds (typically ECD ≥ 2300 cells/mm^2^) restrict the range of transplantable tissue, clinically meaningful variation persists within acceptable parameters.

The relationship between donor age and DMEK outcomes demonstrates a non-linear “donor age paradox.” Young donors (<50 years) typically exhibit higher ECD (>2800 cells/mm^2^) but more elastic tissue that forms tight scrolls (Grade III/IV rolling characteristics), requiring prolonged manipulation and potentially causing 5–15% preparation loss. Conversely, older donors (>65 years) have a lower baseline ECD but stiffer tissue that unrolls more readily (Grade I/II), facilitating “no-touch” techniques with reduced intraoperative trauma. The DROP Score DTQ domain does not simply reward high cell counts but optimizes the balance between biological reserve and surgical handling properties.

The Turkish eye tissue bank infrastructure currently lacks standardization for DMEK tissue preparation. Prestripped and preloaded grafts are unavailable; instead, surgical teams prepare grafts on site in the operating room [33]. This practice introduces variability in effective tissue quality, being dependent on surgeon and center factors not systematically documented in our dataset. Future Turkish DMEK registries should prioritize the collection of detailed tissue preparation variables to enable full DTQ domain utilization.

### 5.8. Strengths and Limitations

#### 5.8.1. Strengths

The DROP Score model has several notable strengths. First, it is based on a comprehensive literature review that identified key factors influencing DMEK outcomes in multiple domains. Clinical validation with real patient data confirms that the model captures significant determinants of surgical outcomes.

Second, the mathematical framework is transparent and interpretable, with clear formulas to calculate component subscores. This transparency allows users to understand how different factors contribute to the overall assessment of risk and facilitates future refinements.

Third, clinical validation demonstrated statistically significant correlations with both BCVA (p=0.007) and ECD (p=0.002), confirming that the DROP Score captures important prognostic information. Identifying diabetes and hypertension as strong risk factors provides actionable clinical insights.

Fourth, temporal analysis revealed healing patterns consistent with the established DMEK physiology, supporting the biological plausibility of our outcome measures and follow-up protocols.

#### 5.8.2. Limitations

This study has several limitations which can be summarized into the following three categories:

**Study design limitations:**

1. The retrospective–prospective hybrid design introduces potential selection bias, as patients with complete 12-month follow-up and adequate IVCM imaging may represent a more compliant or geographically accessible subpopulation.
2. Single-center design limits generalizability across different surgical techniques, eye tissue bank protocols, and patient populations.
3. The absence of a control group or comparator model prevents direct assessment of whether DROP Score-guided management improves outcomes compared to standard care.

**Sample size limitations:**

1. The sample size (*n* = 76) provides only 80% power to detect odds ratios ≥3.0 for binary predictors at α = 0.05, potentially missing smaller but clinically meaningful associations.
2. Only six patients (7.9%) were classified as High Risk, precluding robust statistical comparison between risk categories and preventing meaningful analysis of the Low Risk and Very High Risk categories.
3. The events-per-variable ratio for multivariate analysis (27 poor prognosis events/4 predictors = 6.75) falls below the recommended threshold of 10, increasing the risk of model overfitting.
4. Zero graft failure events during the study period prevented validation of the Cox proportional hazards survival component, limiting graft survival conclusions to synthetic validation only.

**Measurement limitations:**

1. Prognosis classification relied on clinical judgment rather than pre-specified objective criteria, potentially introducing inter-observer variability. Future studies should incorporate formal inter-rater reliability assessment.
2. Default mid-range values for DTQ and CPF subscores, necessitated by incomplete documentation, effectively reduced the model to two active domains (PRP and SCI), limiting assessment of the full four-domain framework’s performance.

### 5.9. Future Directions

Future directions include (i) multicenter validation in diverse populations; (ii) prospective evaluation of DROP Score-guided interventions; (iii) the integration of additional prognostic variables using machine learning approaches; and (iv) the development of user-friendly clinical implementation tools.

## 6. Conclusions

In this single-center clinical validation study, the DROP Score demonstrated statistically significant correlations with 12-month visual acuity (*r* = 0.305, *p* = 0.007) and endothelial cell density (*r* = −0.352, *p* = 0.002) in 76 consecutive DMEK patients. High Risk patients showed numerically higher poor prognosis rates compared to moderate-risk patients (50.0% vs. 34.3%), though this difference did not reach statistical significance due to the small High Risk sample size (*n* = 6). Diabetes mellitus emerged as a statistically significant prognostic factor (OR 4.34, *p* = 0.012) in univariate analysis, though significance was attenuated in multivariate modeling.

These findings should be interpreted within the context of several important limitations: single-center design with restricted risk category distribution (92.1% Moderate Risk), reliance on default values for two of four model domains, subjective prognosis classification, and the absence of graft failure events precluding survival model validation. The modest discrimination (AUC 0.647) and decision curve analysis suggesting comparable performance to simpler models indicate that the clinical utility of the complete DROP Score framework remains to be established.

External validation in multicenter cohorts with complete four-domain data collection, objective outcome definitions, and adequate sample sizes across all risk categories is required before the DROP Score can be recommended for routine clinical use. Prospective studies evaluating whether DROP Score-guided management improves patient outcomes compared to standard care are warranted.

## Figures and Tables

**Table 1:** DROP Score risk stratification categories.

Risk Category	DROP Score	Clinical Interpretation	Recommended Action
Low Risk	0–25	Excellent prognosis	Standard follow-up
Moderate Risk	26–50	Good prognosis with monitoring	Enhanced surveillance
High Risk	51–75	Guarded prognosis	SF6 tamponade, frequent visits
Very High Risk	76–100	Poor prognosis	Consider alternative procedure

**Table 2:** Sensitivity analysis results.

Weight Scenario	Mean	SD	High Risk (%)
Original (0.40, 0.25, 0.20, 0.15)	46.5	5.8	27.6
Equal weights	44.6	4.4	11.8
PRP dominant (0.60)	48.5	8.2	39.5

**Table 3:** Outcomes by DROP Score risk category (synthetic validation).

Outcome	Low Risk	Moderate Risk	High Risk	Very High Risk
Graft failure at 1 year	2.8%	6.6%	12.4%	21.7%
Graft failure at 3 years	5.3%	11.9%	19.8%	32.4%
BCVA at 12 months (logMAR)	0.08	0.14	0.22	0.31
ECL at 12 months	32.4%	36.8%	42.3%	49.7%
Rebubbling	12.1%	22.7%	35.6%	48.3%
Rejection	2.3%	5.8%	10.2%	17.9%

**Table 4:** Comparison of DROP Score model predictions with literature benchmarks.

Outcome	DROP Score Model	Literature Benchmark
1-year Graft Survival	93.5%	94–98%
3-year Graft Survival	87.2%	85–95%
5-year Graft Survival	82.0%	75–88%
BCVA at 12 Months (logMAR)	0.15	0.10–0.20
ECL at 12 Months	37.5%	35–40%
Rebubbling Rate	25.3%	18–39%
Rejection Rate	7.2%	5–10%

**Table 5:** DROP Score distribution and component subscores.

Parameter	Clinical (*n* = 76)	Synthetic (*n* = 500)
**DROP Score Distribution**		
Mean ± SD	39.4 ± 7.6	42.3 ± 15.7
Median (IQR)	41.3 (33.4–45.0)	–
Range	26.1–57.4	12.6–87.9
**Risk Category Distribution**		
Low Risk (0–25)	0 (0.0%)	107 (21.4%)
Moderate Risk (26–50)	70 (92.1%)	243 (48.6%)
High Risk (51–75)	6 (7.9%)	121 (24.2%)
Very High Risk (76–100)	0 (0.0%)	29 (5.8%)
**Component Subscores (Mean ± SD)**		
Patient Risk Profile (PRP)	37.3 ± 17.1	–
Donor Tissue Quality (DTQ)	50.0 *	–
Surgical Complexity Index (SCI)	22.2 ± 17.2	–
Center Performance Factor (CPF)	50.0 *	–

* Default mid-range values used due to limited donor/center data availability.

**Table 6:** DROP Score model discrimination.

Outcome	AUC/*r*	95% CI/*p*-Value	Reference *
**Binary Outcomes (AUC)**			
Poor Prognosis (12-month)	0.647	0.520–0.771	0.782
Rebubbling	0.571	0.405–0.713	0.763
**Continuous Outcomes (Pearson’s *r*)**			
12-month BCVA (LogMAR)	0.305	p=0.007	0.684
12-month ECD (cells/mm^2^)	−0.352	p=0.002	0.712
12-month CCT (μm)	0.073	p=0.528	–

* Reference values from synthetic validation dataset (*n* = 500).

**Table 7:** Clinical outcomes by DROP Score risk category.

Outcome	Moderate Risk (*n* = 70)	High Risk (*n* = 6)	*p*-Value
Poor Prognosis (12-mo)	24 (34.3%)	3 (50.0%)	0.420
Rebubbling	12 (17.1%)	0 (0.0%)	0.584
BCVA at 12-mo (LogMAR)	0.31 ± 0.49	0.50 ± 0.31	0.195
CCT at 12-mo (μm)	529 ± 48	560 ± 60	0.127
ECD at 12-mo (cells/mm^2^)	1187 ± 417	1078 ± 376	0.538

**Table 8:** Clinical parameters by follow-up timepoint.

Parameter	3 Months	6 Months	12 Months
BCVA (LogMAR)	0.47 ± 0.50	0.42 ± 0.55	0.33 ± 0.48
CCT (μm)	527.1 ± 59.4	525.5 ± 53.8	531.5 ± 49.4
ECD (cells/mm^2^)	1370 ± 486	1239 ± 452	1178 ± 413
Good Prognosis (%)	56.6%	61.8%	64.5%
ΔLogMAR from Preop	−0.70	−0.75	−0.84

**Table 9:** Multivariate logistic regression for poor prognosis at 12 months (*n* = 72).

Factor	Adjusted OR	95% CI	*p*-Value
Diabetes Mellitus	2.29	0.59–8.90	0.232
Hypertension	2.17	0.60–7.88	0.238
Epithelial Debridement	3.14	0.98–10.05	0.054
PBK (vs. FECD)	2.66	0.90–7.85	0.076

Multivariate logistic regression adjusting for all listed factors. Pseudo $R^{2}$ = 0.139. No factor achieved statistical significance (p<0.05) after adjustment; epithelial debridement showed the strongest independent association.

**Table 10:** Univariate prognostic factors for poor prognosis at 12 months (*n* = 72).

Factor	Odds Ratio	95% CI	*p*-Value
**Systemic Comorbidities**			
Diabetes Mellitus	4.34	1.44–13.14	0.012 *
Hypertension	2.65	0.98–7.15	0.078
**Primary Diagnosis**			
PBK (vs. FECD)	3.00	1.11–8.14	0.051
FECD (protective)	0.33	0.12–0.90	0.051
**Ocular Comorbidities**			
Preoperative Glaucoma	2.27	0.67–7.68	0.214
**Surgical Factors**			
Epithelial Debridement	2.73	0.97–7.74	0.082
SF6 Tamponade	1.13	0.29–4.43	1.000
Rebubbling	0.80	0.22–2.98	1.000
Triple DMEK	0.63	0.20–1.99	0.576

* p<0.05. Fisher’s exact test used for all comparisons. CI = Confidence Interval.

**Table 11:** Comparison of clinical outcomes with DROP score predictions and literature benchmarks.

Outcome	Clinical (*n* = 76)	DROP Prediction	Literature
**Graft Survival Outcomes**			
1-Year Graft Survival	100.0%	93.5%	94–98%
Rebubbling Rate	15.8%	25.3%	18–39%
**Visual Outcomes**			
BCVA at 12-mo (LogMAR)	0.33 ± 0.48	0.15	0.10–0.20
VA Improvement (LogMAR)	0.84	∼0.80	0.70–1.00
**Corneal Parameters**			
CCT at 12-mo (μm)	531.5 ± 49.4	531	500–550
ECD at 12-mo (cells/mm^2^)	1178 ± 413	1987	1500–2000
**Prognosis**			
Good Prognosis (12-mo)	64.5%	∼65%	60–75%

**Table 12:** Comparison of synthetic and clinical validation results.

Parameter	Synthetic (*n* = 500)	Clinical (*n* = 76)
DROP Score Range	12.6–87.9	26.1–57.4
Risk Category Distribution	All 4 categories	Moderate/High only
AUC (Primary Outcome)	0.782	0.647
BCVA Correlation	r=0.684	r=0.305 (p=0.007)
ECD Correlation	r=0.712	r=−0.352 (p=0.002)
1-Year Graft Survival	93.5%	100%
Rebubbling Rate	25.3%	15.8%

**Table 13:** Comparative ECD outcomes by diagnosis.

Metric	FECD Benchmark	PBK Clinical	DROP Implication
5-Year Survival	∼98%	∼64%	Diagnosis-specific survival curves
12-Month ECL	∼30–35%	∼45–60%	λ coefficient 1.4–1.6× higher for PBK
Rebubbling Rate	∼10–20%	∼30–50%	Higher SCI weight for complex anatomy

**Table 14:** Model Comparison Statistics.

Model	AUC	Brier	AIC
Intercept Only	0.500	0.234	97.3
DM Only	0.644	0.211	92.2
DM + PBK	0.705	0.202	91.2
Full Model	0.751	0.189	90.6

## Data Availability

The datasets generated and/or analyzed during the current study are not publicly available due to the privacy of the patients involved in this study, but are available from the corresponding author on reasonable request.
